# Applications of Olefin Metathesis in the Synthesis of Fluorinated Substrates and Design of Fluorinated Catalysts

**DOI:** 10.1007/s41061-025-00532-w

**Published:** 2025-11-10

**Authors:** Anas Semghouli, Santos Fustero, Loránd Kiss

**Affiliations:** 1 Institute of Organic Chemistry, Stereochemistry Research Group, HUN-REN Research Center for Natural Sciences, Magyar Tudósok Krt. 2, Budapest, 1117 Hungary; 2 Department of Organic Chemistry, Pharmacy Faculty, University of Valencia, 46100 Burjassot Valencia, Spain

**Keywords:** Catalysis, Fluorine-containing catalysts, Metathesis, Olefin functionalization, Organofluorine compounds

## Abstract

As a result of the high pharmaceutical relevance of organofluorine compounds in drug discovery, the synthetic approach towards this class of derivatives has generated increasing interest in organic chemistry over the past decade. Metathesis, with the manipulation of the C = C double bonds, is considered to be a powerful tool in preparative organic chemistry to access various sophisticated and densely functionalized scaffolds with olefin bonds in their structure. The current paper is intended to describe, investigate, and analyze the most impactful advances and applications of metathesis with organofluorine molecular entities achieved since the outstanding review by Fustero, Haufe and others (Chem. Rev. 2015, 115, 871 − 930, dx.doi.org/10.1021/cr500182a) published a decade ago.

**Graphical abstract:**

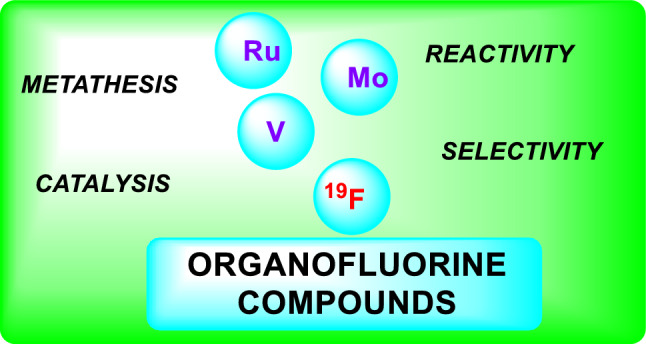

## Introduction

Organofluorine compounds have gained significant attention across various scientific domains, including pharmaceuticals [1–8], agrochemicals [9–11], and materials science [12], thanks to the unique traits that fluorine atoms bring to the table. When fluorine is incorporated into organic molecules, it can significantly alter their physical and chemical properties, like lipophilicity, metabolic stability, and electronic characteristics. This often results in enhanced bioactivity in drug discovery and improved performance in industrial applications. The carbon–fluorine (C–F) bond is particularly noteworthy, boasting a bond strength of about 105.4 kcal/mol, making it the strongest single bond that carbon can form, contributing to the exceptional thermal and chemical stability of fluorinated compounds. Moreover, fluorine has a high electronegativity (EN = 3.98) and a small atomic radius (1.47 Å), allowing it to modulate acidity, basicity, and hydrogen-bonding capabilities within molecular frameworks. These qualities have led to the widespread incorporation of fluorinated motifs in many of the pharmaceutical drugs we discover nowadays [13–18]. Furthermore, there are two main strategies to introduce fluorine into complex organic molecules. The first method involves direct connection of fluorine atoms or fluorinated groups (like CF₃) to an organic framework using nucleophilic or electrophilic fluorinating agents [19]. The second method uses fluorinated building blocks that are incorporated during a synthetic sequence. This review will mainly focus on the latter approach [20].

**Fig. 1:**
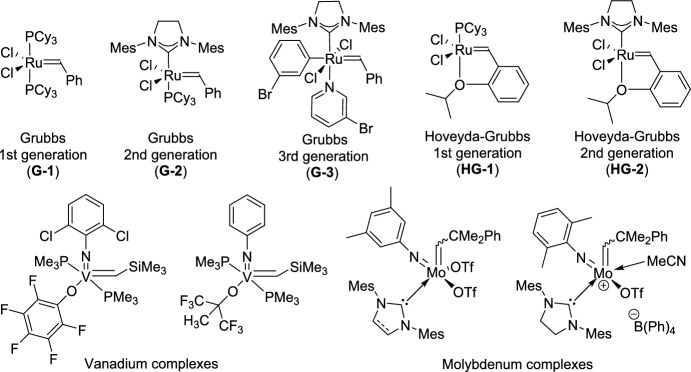
Some representative vanadium-, molybdenum-, and ruthenium-based catalysts discussed in this review

Olefin metathesis is a powerful catalytic process that allows the breaking and reformation of carbon–carbon double bonds in alkenes, leading to the creation of new olefinic structures. The reaction utilizes well-defined metal alkylidene complexes, mainly those based on ruthenium, molybdenum, or vanadium, which are known for their impressive tolerance to different functional groups and stability even under harsh conditions (Fig. 1) [21–24]. Olefin metathesis has revolutionized organic synthesis, particularly in the formation of carbocycles and heterocycles through ring-closing metathesis (RCM). Other variations, including cross-metathesis (CM), ring-opening metathesis polymerization (ROMP), and acyclic diene metathesis polymerization [25], further expand its synthetic utility. Beyond small-molecule synthesis, metathesis plays a crucial role in material science, polymer chemistry, and the development of bioactive molecules, making it one of the most versatile and significant transformations in modern organic chemistry [26].

This review aims to describe and analyze the most significant advancements in metathesis involving the synthesis of fluorinated derivatives since the landmark paper by Haufe et al. [20f]. We discuss key developments in RCM, CM, and ROMP, along with progress in fluorine-containing catalysts. These advances demonstrate the crucial role of fluorine in enhancing reactivity, selectivity, and catalyst performance.

## Ring-Closing Metathesis (RCM) with Fluorinated Derivatives

Among the several existing categories within metathesis reactions, RCM has been the most frequently used in synthetic organic chemistry. The main reason is that cyclic molecules are key elements in fields such as natural products, medicinal chemistry, and materials science.

**Scheme 1:**
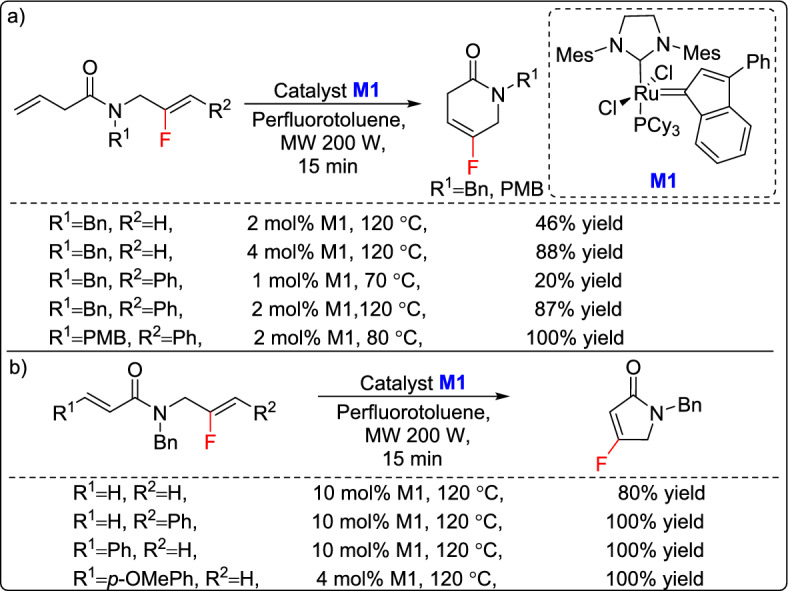
Synthesis of (a) five- and (b) six-membered fluorinated lactams

In 2014, Couve-Bonnaire and co-workers reported a novel approach for the homogeneous RCM of challenging fluorinated compounds through microwave irradiation, achieving high reactivity and yield for both five- and six-membered fluorinated lactams [27]. The key to this success was the strategic combination of a fluorinated aromatic solvent and appropriate double-bond substitution, which significantly influenced electron density and catalytic efficiency. Notably, six-membered ring formation proceeded efficiently, even with minimal catalyst loading and reduced temperatures. The study also highlighted the role of electron-donating and electron-withdrawing groups on phenylated fluoroalkenes, showing that electron-donating groups enhanced the catalytic process (Scheme 1a). Additionally, they successfully created five-membered rings, demonstrating the importance of substrate modifications beyond just the least reactive olefin (Scheme 1b).

**Scheme 2:**
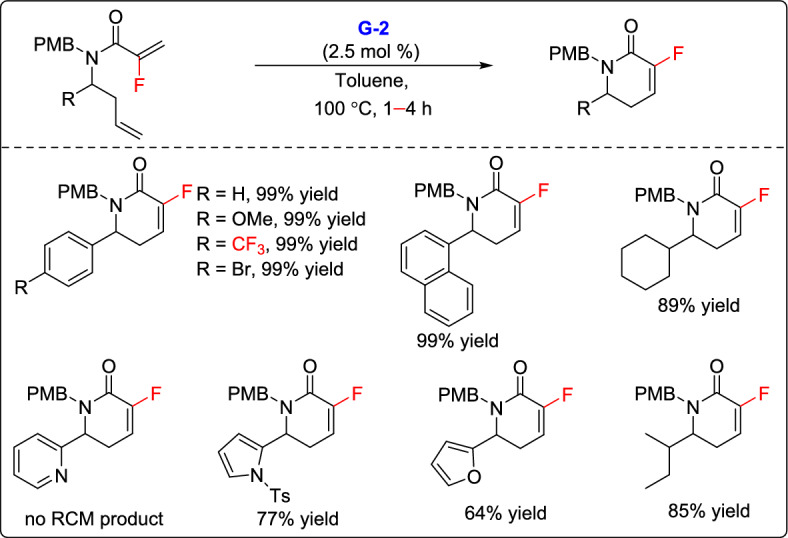
Synthesis of diverse fluorinated δ-lactams

In 2015, Marquez and co-workers reported an efficient strategy for the synthesis of a series of fluorinated δ-lactams, which could serve as new building blocks in medicinal chemistry [28]. Their methodology involved an RCM reaction of fluorinated olefins bearing an amide functionality protected with a *para*-methoxybenzyl group, with diverse α-substituents at the nitrogen atom. By employing 2.5 mol% of the second-generation Grubbs catalyst (G-2), a variety of substrates featuring aryl, bromoaryl, alkyl, furyl-, and tosyl-protected pyrrole substituents underwent smooth cyclization to afford the desired fluorinated lactams in very high yields up to 99% (Scheme 2). This work established a solid basis for creating new fluorinated ring systems, and ongoing efforts are focused on expanding the scope to include pyridines and pyrroles, as well as exploring enantioselective variants of this strategy.

**Scheme 3:**
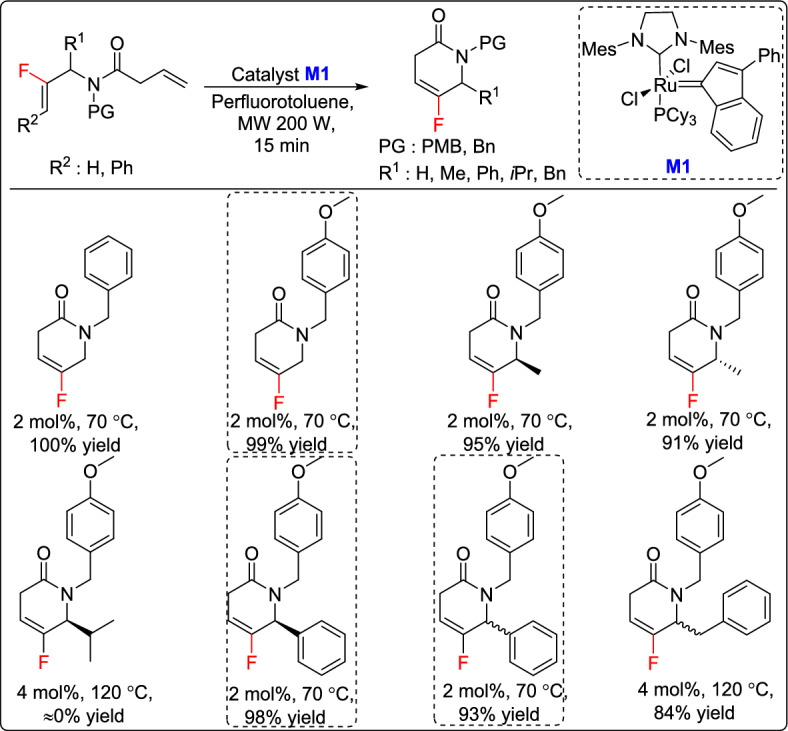
Synthesis of fluoroalkene-containing lactams

One year later, the same research group led by Couve-Bonnaire reported an efficient approach for accessing constrained fluoropseudopeptides via RCM of fluoroalkenes. The study began with the synthesis of bis-alkene substrates with a fluoroalkene unit linked by an amide moiety, which were then subjected to the RCM protocol. Several ruthenium precatalysts were tested and the M1 catalyst proved to be the most efficient for producing fluorinated lactams in high yields, except in the case of a hindered substrate bearing an isopropyl group as a side chain. In this case, the reaction was unsuccessful regardless of catalyst loading or reaction temperature, yielding only side products (Scheme 3). Furthermore, three of the synthesized lactams were deprotected and subjected to ring-opening reactions, offering a novel route to constrained fluoropseudopeptides bearing a fluoroalkene moiety as a peptide bond mimic [29].

**Scheme 4:**
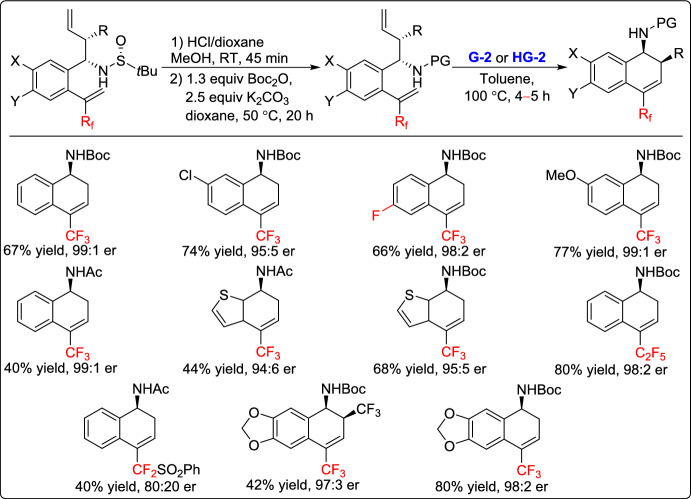
Synthesis of fluorinated benzo-fused bicycles

In the same year, Fustero and co-workers developed a novel approach for the synthesis of enantiomerically-enriched fluorinated benzo-fused bicyclic homoallylic amines through an asymmetric allylation/RCM sequence. This strategy begins with the synthesis of *α*-trifluoromethylstyrene derivatives as key intermediates. Then these were subjected to the RCM reaction in the presence of 5 mol% of the G-2 catalyst in toluene at 100 °C, leading to the bicyclic derivatives with moderate to good yields (40–80%) and high enantioselectivity (Scheme 4). The study demonstrated that *α*-fluoroalkylated styrenes exhibit significantly lower reactivity in RCM compared to their nonfluorinated counterparts. This work introduces a new family of fluorinated building blocks for the synthesis of more complex molecules, paving the way for further applications of the *α*-CF_3_-substituted 2-vinylbenzaldehyde scaffold [30].

**Scheme 5:**
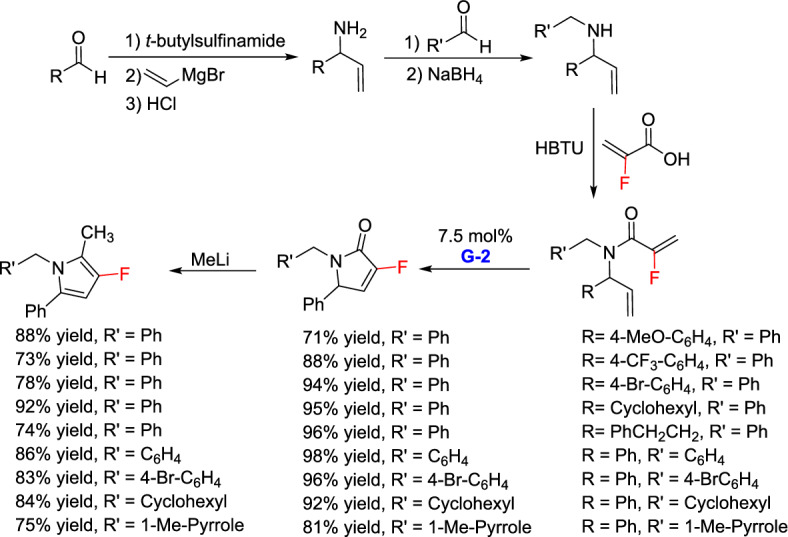
Synthesis of polyfunctionalized 3-fluoropyrroles

In the same year, a new and efficient approach for the synthesis of polyfunctionalized 3-fluoropyrroles was reported by Marquez and co-workers, providing an alternative and highly convergent strategy for assembling these chemically and biologically significant molecules [31]. The methodology started from commercially available aldehydes and involved a concise sequence of transformations, each delivering products with high yields and excellent selectivity. It began with the condensation of various aldehydes with *tert*-butylsulfinamide forming the corresponding imine which, upon vinylation with vinyl magnesium bromide, produced the allylic amine. Reductive amination of the amine formed with substituted aldehyde afforded the Bn-protected amine, which was subsequently coupled with 2-fluoroacrylic acid to generate the desired amide unit. Treatment of the resulting diene with the G-2 catalyst led to the formation of *α*,*β*-unsaturated lactam. Finally, alkylation with methyllithium cleanly furnished the target 3-fluoropyrrole in yields of 73–92% (Scheme 5).

**Scheme 6:**
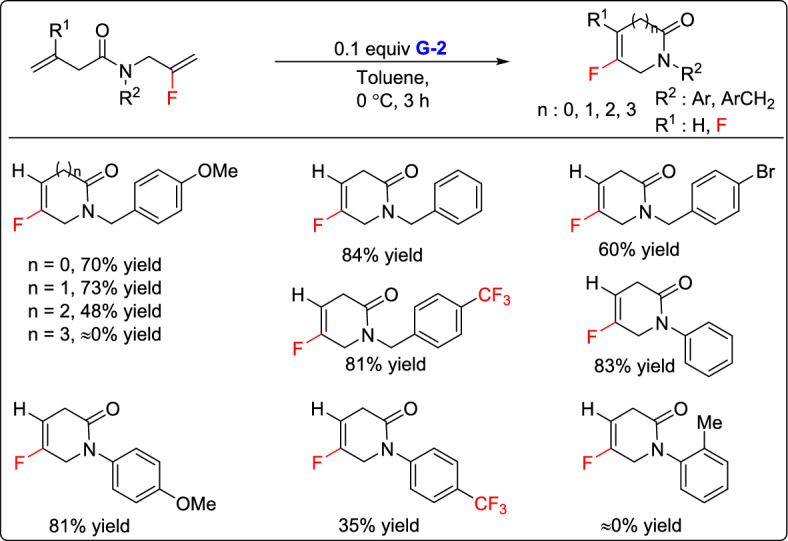
Synthesis of fluoroalkene-containing lactams

In 2016, Lei and co-workers reported a cost-effective approach for synthesizing a range of *N*-substituted 2-fluoroallylamines, and demonstrated their application in the preparation of fluoroalkene-containing lactams [32]. The method involved the synthesis of *N*-substituted 2-fluoroallylamines from methyl 2-fluoroacrylate via aminolysis and subsequent selective reduction of the amide group. These amines were then coupled with various alkenoic acids, and the resulting amide intermediates underwent RCM in the presence of 0.1 equivalents G-2 catalyst leading to the formation of fluorovinyl-containing lactams in good yields. The five- and six-membered ring products were obtained in high yields (70% and 73%, respectively), whereas the seven-membered ring formed with a lower yield (48%), and an eight-membered ring was not produced. Substrates with an *ortho* methyl group on the benzene ring failed to undergo RCM. Instead, *α*,*β*-unsaturated amides were formed via a 1,3-H shift, suggesting that the *ortho* methyl group may hinder the cyclization process (Scheme 6).

**Scheme 7:**
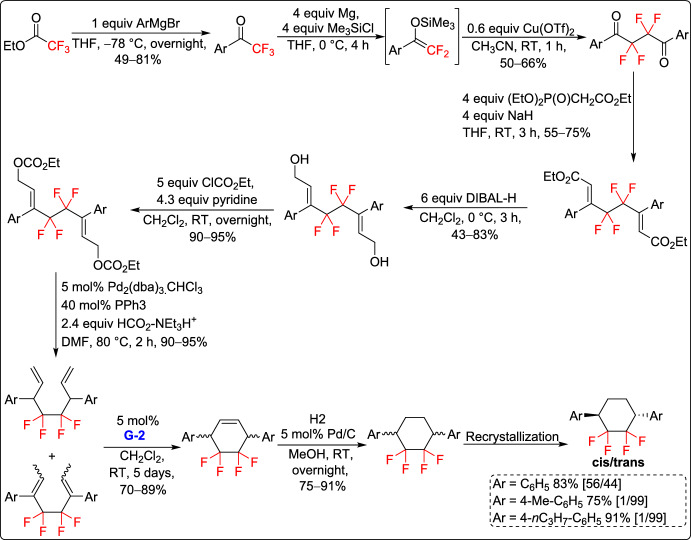
Synthesis of symmetrical 3,6-disubstituted 1,1,2,2-tetrafluorocyclohexane derivatives

**Scheme 8:**
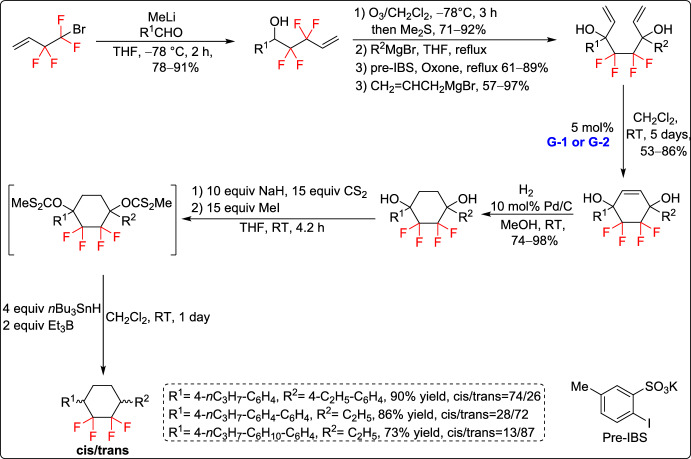
Synthesis of asymmetrical 3,6-disubstituted 1,1,2,2-tetrafluorocyclohexane derivatives

Konno and co-workers introduced a new method for synthesizing 3,6-disubstituted 1,1,2,2-tetrafluorocyclohexane derivatives in 2017, starting from readily available fluorinated materials. They established effective synthetic procedures for both symmetric and non-symmetric derivatives, enabling the preparation of these unique fluorinated carbocycles using RCM as the key step for the construction of the six-membered ring core from acyclic precursors (Schemes 7 and 8). Notably, some of the obtained tetrafluorinated cyclohexanes underwent recrystallization, resulting in *trans*-configured products with high selectivity (Scheme 7). Among them, a *trans*-disubstituted tetrafluorocyclohexane derivative exhibited remarkable properties, such as low birefringence (Δn = 0.073) and a strong negative dielectric anisotropy (Δε = – 9.4) in a binary mixture system. These characteristics make it a promising material for VA-mode liquid crystal (LC) displays. These findings offer important guidance for the design of novel LC molecules with optimized optical and electronic properties for high-performance display technologies [33].

**Scheme 9:**
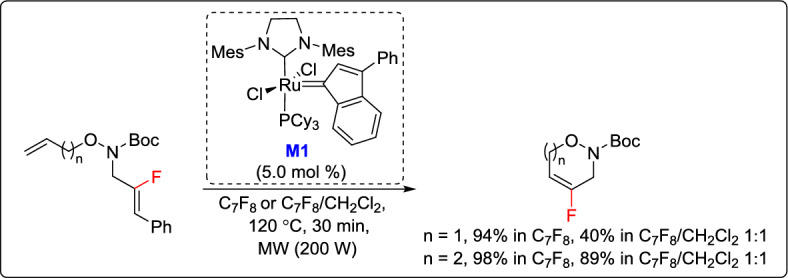
Synthesis of fluorinated 1,2-oxazine and 1,2-oxazepine

In the same year, Couve-Bonnaire and his team developed an efficient method to synthesize fluorinated 1,2-oxazine and 1,2-oxazepine derivatives, expanding the scope of their RCM-based methodology toward novel fluorinated heterocycles [34]. The approach relies on the use of the M2 precatalyst in perfluorinated toluene to mediate the RCM of trisubstituted olefins, delivering 4-fluoro-3,6-dihydro-1,2-oxazine and 4-fluoro-3,6,7-trihydro-1,2-oxazepine in nearly quantitative yields. Despite the high efficiency, the method required highly diluted conditions to avoid homodimerization side reactions, leading to excessive use of the costly perfluorinated solvent. To overcome this limitation, the authors optimized the process by using a mixed solvent system composed of dichloromethane and octafluorotoluene (1:1), significantly reducing solvent consumption while keeping satisfactory yields (Scheme 9).

**Scheme 10:**
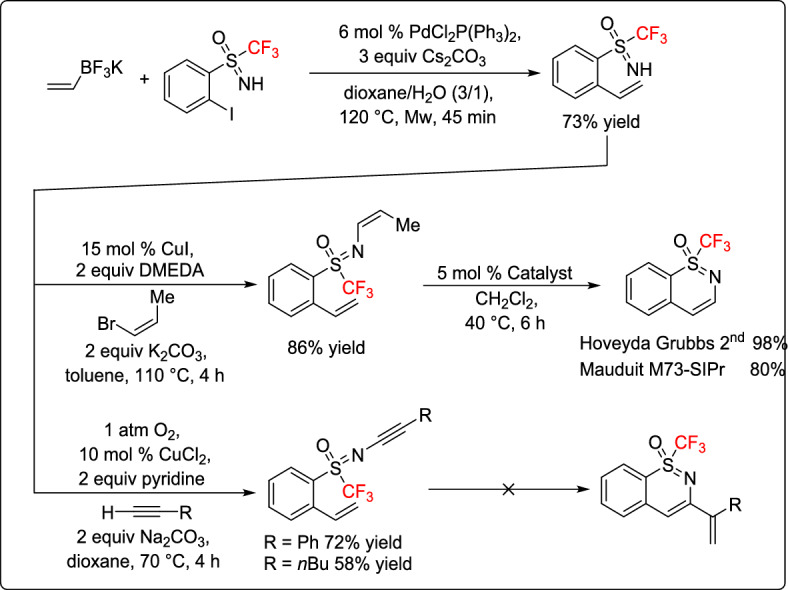
Synthesis of fluorinated cyclic sulfoximines

Magnier and co-workers reported a novel approach for the preparation of *ortho*-vinylaryl *S*-trifluoromethylated sulfoximines through a palladium-catalyzed cross-coupling and metathesis rearrangement sequence [35]. They successfully used Stille and Suzuki–Miyaura coupling methodologies to convert *ortho*-iodo aryl sulfoximines into various vinyl derivatives in good yields. The resulting fluorinated derivatives exhibited distinct reactivity compared to their nonfluorinated counterparts, enabling the use of free NH sulfoximines in further coupling processes. Finally, metathesis transformations were explored after post-functionalization of the nitrogen atom with a vinyl group, ultimately leading to the formation of previously unknown cyclic sulfoximines (Scheme 10). These findings highlight the potential of fluorinated sulfoximines as valuable building blocks for further synthetic applications.

**Scheme 11:**
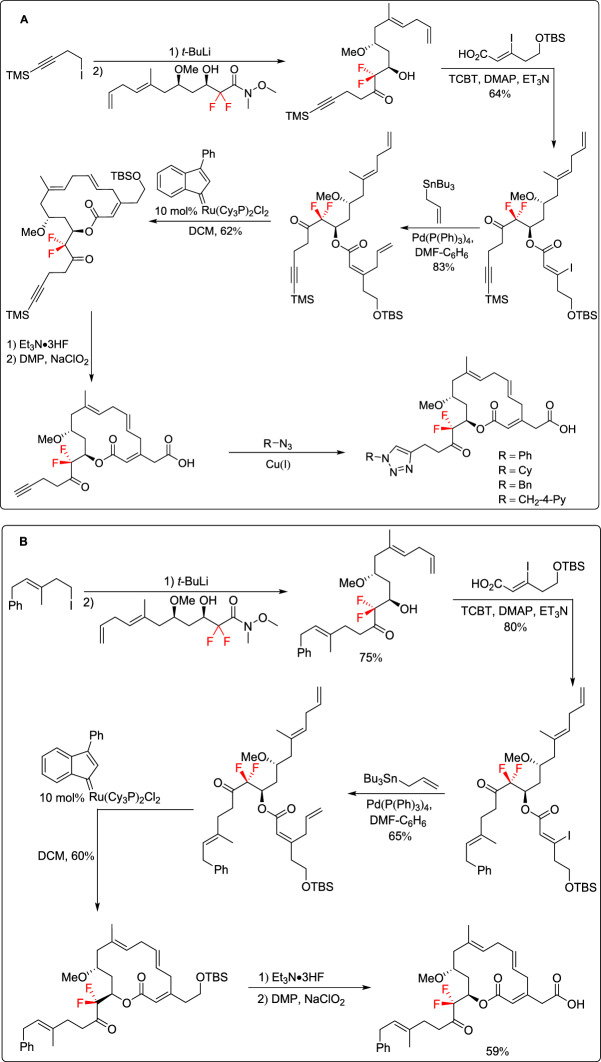
(A, B) Synthesis of analogues of ripostatin A (TCBT = 2,4,6-trichlorobenzoyl chloride)

In 2019, Shenderman and Prusov developed a methodology for the synthesis of fluorinated analogues of ripostatin A, featuring heterocyclic side-chain modifications. The process began with the reaction of a Weinreb amide and an organolithium reagent generated from iodide or via metal/halogen exchange, affording hydroxyketones in good yield (Scheme 11). Next, the iodoacrylic acid fragment was attached using a modified Yamaguchi procedure. The second allyl group was installed into the acrylate moiety through a Stille cross-coupling reaction, and the macrolactones were formed through a RCM process using an indenyl-based catalyst developed by Fürstner. The protecting groups (TBS or TMS) were removed through sequential treatment with TBAF and the Et_3_N 3HF complex. A two-step oxidation protocol yielded 11-O-Me-14,14’-difluororipostatin A (Scheme 11A) and the penultimate acid (Scheme 11B). The final step involved Cu-catalyzed cycloaddition reactions with various azides, producing triazole-containing analogues of ripostatin A (Scheme 11). The resulting compounds, although less potent than ripostatin B derivatives, exhibited moderate activity against pathogenic micro-organisms, underscoring their potential as candidates for further structure–activity relationship studies [36]. 11*-O*-Me-14,14’-difluoro-ripostatin A was synthetized following similar reaction sequencies, and, finally, by deprotection of the protected TBS ether, and two oxidations steps provided the desired target derivative (Scheme 11).

**Scheme 12:**
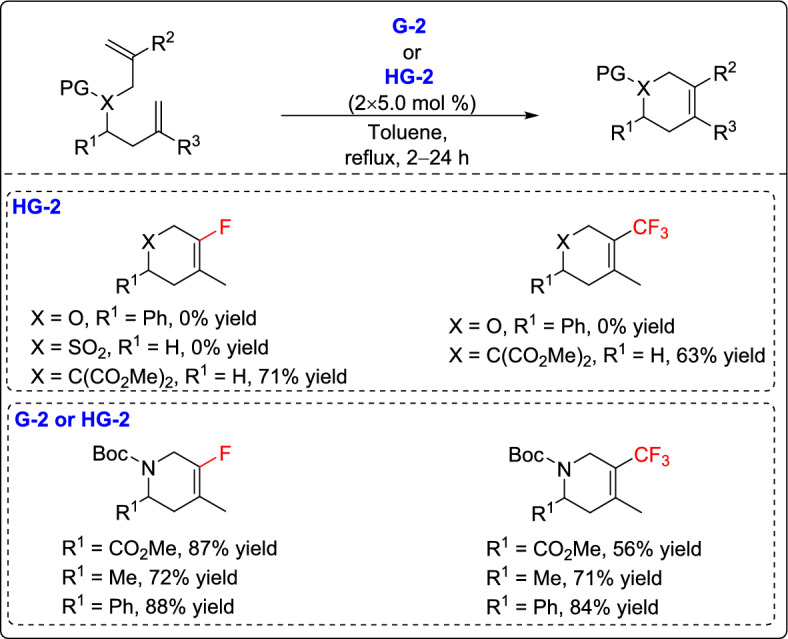
Synthesis of fluorinated tetrahydropyridines

In 2020, Michelet and co-workers introduced a practical and efficient approach for the synthesis of fluorinated tetrahydropyridines via RCM [37]. The methodology employed tetrasubstituted dienes bearing fluoroalkene moieties catalyzed by the second-generation Hoveyda–Grubbs catalyst (HG-2), which demonstrated superior performance compared to G-2. This catalytic system enabled the successful formation of a series of distinct fluorinated tetrahydropyridines in good yields(13). However, the approach showed limitations, as it failed to create *O*-heterocycles and cyclic sulfones under the same conditions. Despite this, the study offered valuable insights into the behavior of tetrasubstituted olefins in RCM, and the resulting tetrahydropyridines show great potential as useful intermediates in drug development (Scheme 12).

**Scheme 13:**
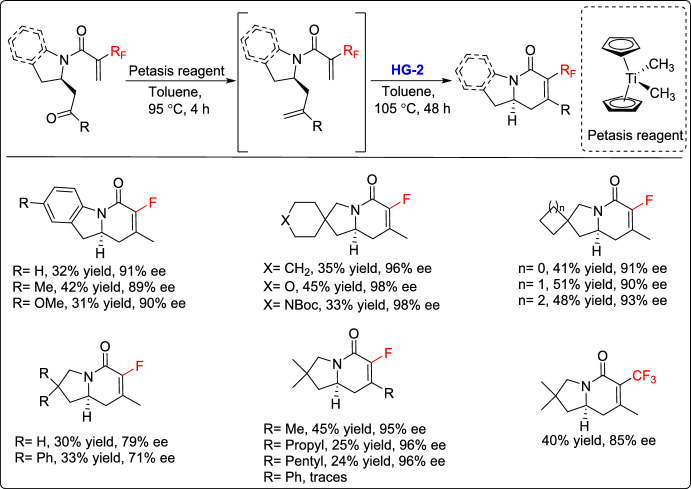
Synthesis of fluorinated indolizidine derivatives by the methylenation/RCM sequence

Recently, Escolano and coworkers reported an enantioselective approach for the synthesis of a diverse range of fluorinated indolizidinone derivatives via a sequence of reactions involving aza-Michael addition, methylenation, and RCM [38]. The procedure involved conjugated fluorinated amides bearing a pendant *α*,*β*-unsaturated ketone moiety, which were subjected to an intramolecular aza-Michael reaction catalyzed by (*S*)-TRIP-derived phosphoric acid to generate intermediates with high enantioselectivity. Subsequent carbonyl methylenation using the Petasis reagent and RCM with the HG-2 catalyst delivered fluorinated indolizidinone derivatives featuring a tetrasubstituted double bond. This method preserved the stereochemical center throughout the synthetic pathway, resulting in moderate yields with excellent enantioselectivities (Scheme 13).

**Scheme 14:**
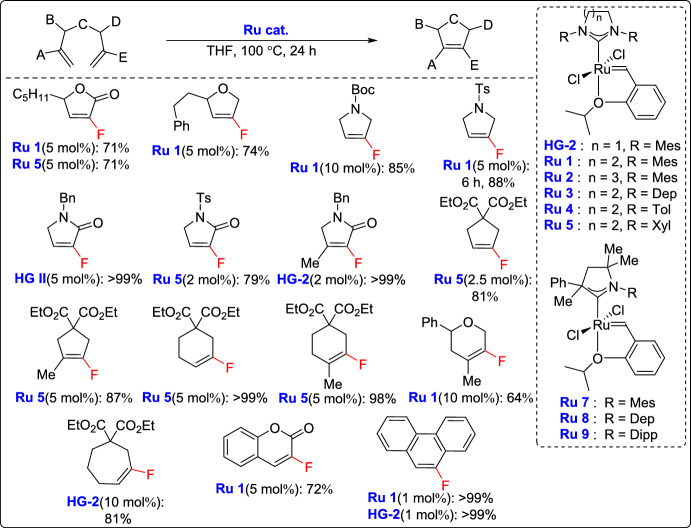
Synthesis of five- and six-membered fluorinated cyclic olefins

In a recent study, Inoue and colleagues introduced an efficient strategy for the synthesis of five- and six-membered fluorinated cyclic olefins, including carbocycles, ethers, lactones, and nitrogen-containing rings, through RCM using ruthenium catalysts bearing six-membered *N*-heterocyclic carbene (NHC) ligands [39]. The methodology proved to be especially effective for creating cyclic ethers, which are usually difficult to produce with traditional catalysts. The enhanced reactivity was attributed to the strong electron-donating nature of the NHC ligands, which facilitated the formation of the key metallacyclobutane intermediate. Additionally, the steric bulk of the ligand influenced its cleavage, as confirmed by DFT calculations. This approach enabled the synthesis of a wide range of fluorinated olefinic rings in good yields, expanding the scope of RCM in the construction of fluorinated ring systems (Scheme 14).

## Cross-Metathesis (CM) with Fluorinated Derivatives

CM, another important type of metathesis reaction, is frequently applied in synthetic organic chemistry to create new carbon–carbon double bonds. This reaction is particularly valuable because of its ability to selectively exchange alkenes, making it a powerful tool for synthesizing diverse and complex molecules in fields like medicinal chemistry, agrochemicals, and materials science.

**Scheme 15:**
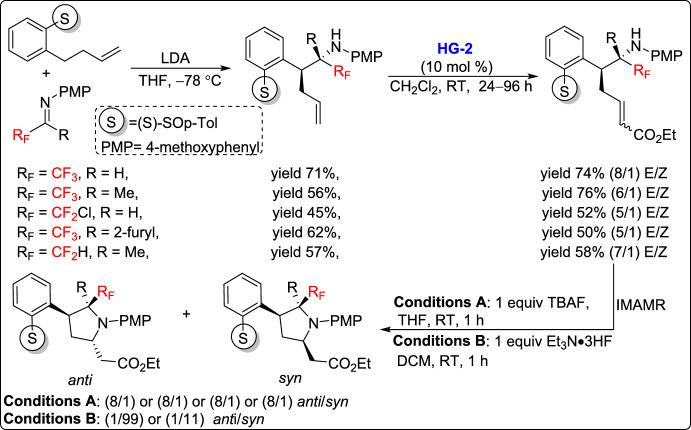
Synthesis of cyclic fluorinated β^3^-amino acid derivatives via CM-IMAMR

In 2015, Fustero and co-workers reported a novel strategy for the stereocontrolled synthesis of fluorinated homoproline derivatives via a tandem CM and intramolecular aza-Michael reaction (IMAMR). The key to this approach was the use of 2-*p*-tolylbenzyl carbanions as chiral benzylic nucleophiles, enabling highly diastereoselective addition to fluorinated imines, producing amines as single diastereoisomers. The resulting intermediates underwent CM reaction with ethyl acrylate in dichloromethane at room temperature, yielding conjugated esters in moderate to good yields with high selectivity, achieving *E*/*Z* diastereoisomeric ratios ranging from 5:1 to 8:1. This was followed by IMAMR, efficiently forming cyclic β^3^-amino acid derivatives (Scheme 15). Notably, a stereodivergent cyclization was achieved by a simple modification of the reaction conditions, allowing selective access to distinct diastereomers. Furthermore, the influence of the sulfoxide auxiliary on the stereochemical outcome was systematically evaluated, providing insight into the factors governing selectivity in this transformation [40].

**Scheme 16:**
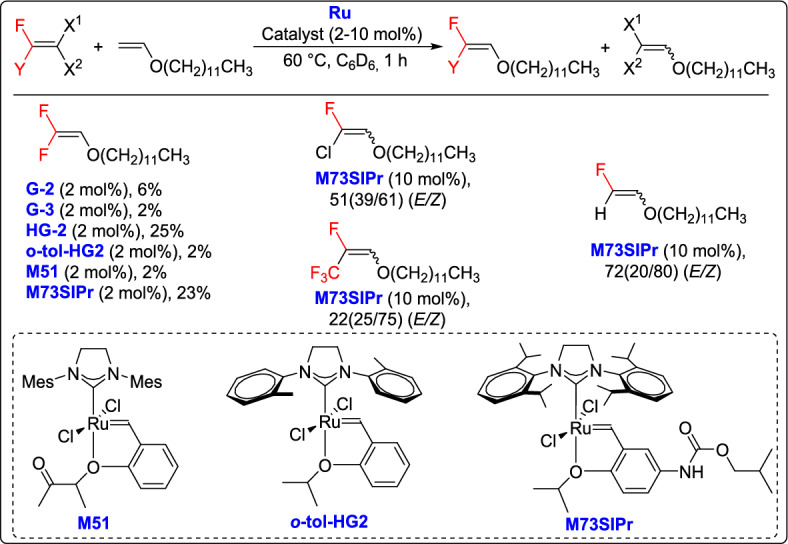
Synthesis of fluorinated olefins with multiple fluorine atoms (Y = F, H, Cl, CF_3_)

Takahira and Morizawa described an approach for the ruthenium-catalyzed olefin CM with tetrafluoroethylene (TFE) and its analogues, demonstrating the feasibility of synthesizing a new class of partially fluorinated olefins of high synthetic value [41]. It was found that ruthenium precatalysts bearing a (2-isopropoxyphenyl)methylidene moiety exhibited enhanced catalytic activity for this transformation. The absence of phosphane ligand was considered to be a key factor contributing to the superior results. In contrast, G-2, fast-initiating G-3, and sterically less-hindered o-tol-HG-2 were not effective for this reaction. Notably, not only TFE but also analogous fluoroolefins participated effectively in this transformation. Using the M73SIPr precatalyst, these fluoro-olefins were converted under mild reaction conditions, affording the corresponding products in moderate to good yields (Scheme 16). This breakthrough offered a simple and highly effective way to create fluorinated olefins with multiple fluorine atoms, which are of great significance in organic synthesis, medicinal chemistry, and materials science.

**Scheme 17:**
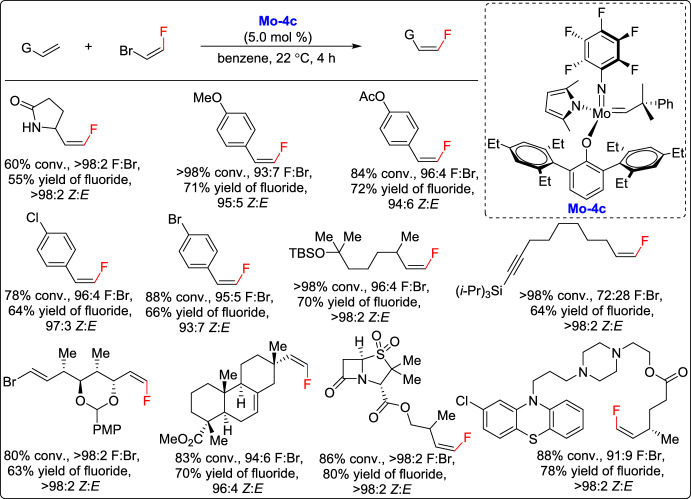
Synthesis of *Z*-alkenyl-fluorides through catalytic CM

One year later, Hoveyda and co-workers introduced a groundbreaking approach for the synthesis of acyclic 1,2-disubstituted *Z*-alkenyl halides via olefin metathesis, significantly broadening the range of halogenated alkenes that could be produced through catalytic processes. This method relied on previously unknown halo-substituted molybdenum alkylidene species, which demonstrated exceptional reactivity and efficiency in promoting high-yielding cross-metathesis reactions. A key feature of this strategy was the use of minimal amounts of the in situ-generated catalyst Mo-4c in combination with commercially available liquid 1,2-dihaloethene reagents. The transformations proceeded at ambient temperature within 4 h, delivering alkenyl fluorides in yields up to 80% with complete *Z* selectivity (Scheme 17). Importantly, this method enabled the site- and stereoselective fluorination of complex organic molecules, offering a practical and cost-effective alternative to expensive and cumbersome fluoro-olefin reagents. These findings established a powerful synthetic tool for accessing fluorinated and halogenated alkenes, offering valuable applications in medicinal chemistry and materials science [42].

**Scheme 18:**
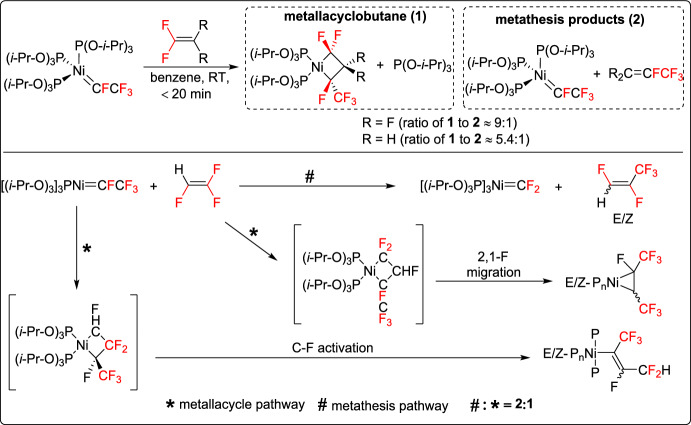
Nickel fluorocarbene metathesis with fluoroalkenes

Baker and his team developed a new method for nickel fluorocarbene metathesis with fluoroalkenes [43]. The study focused on a nickel tris(phosphite) fluoro(trifluoromethyl)carbene complex ([P_3_Ni] = CFCF_3_), which reacted with TFE and vinylidene fluoride to yield both metallacyclobutane and perfluorocarbene metathesis products, namely [P_3_Ni] = CF_2_ and CR_2_ = CFCF_3_ (R = F, H). Interestingly, reactions with TFE led to distinct products, including [P_3_Ni] = CF_2_ and *cis*/*trans*-CFCF_3_ = CFH, due to the formation of metallacyclopropanes and fluoronickel alkenyl species rather than conventional metallacyclobutanes. DFT calculations and experimental studies revealed that the observed metallacyclobutanes were not intermediates in metathesis product formation, highlighting a novel mechanistic pathway distinct from the classical Chauvin mechanism, driven by unique four-coordinate intermediates (Scheme 18).

**Scheme 19:**
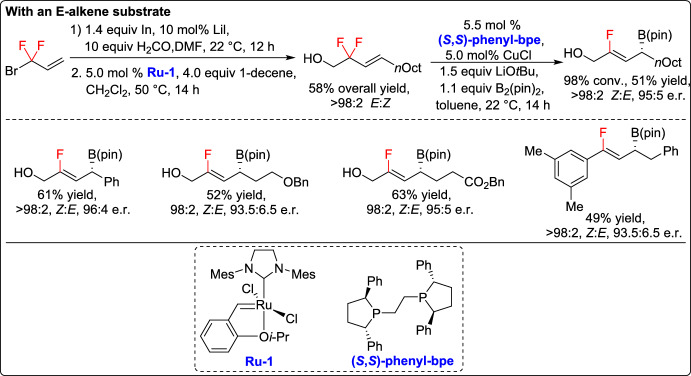
Synthesis of allylic boronates bearing a *Z*-trisubstituted alkenyl fluoride

In 2019, Ito and co-workers developed a groundbreaking catalytic method for the diastereo- and enantio-selective synthesis of allylic boronates bearing a *Z*-trisubstituted alkenyl fluoride [44]. This approach utilized bisphosphane/Cu complexes to catalyze boryl substitution of both *Z*- and *E*-allyldifluorides, achieving the desired products in up to 99% yield with exceptional stereoselectivity (> 98:2 *Z*/*E* selectivity and 99:1 enantiomeric ratio). Furthermore, the resulting allylic boronates proved highly versatile, allowing diverse post-functionalization strategies. Notably, diastereoselective additions to aldehydes and aldimines facilitated the construction of homoallylic alcohols and amines featuring a fluorosubstituted stereogenic quaternary center, expanding the synthetic toolkit for the preparation of complex fluorinated molecules with accurate stereochemical control (Scheme 19).

**Scheme 20:**
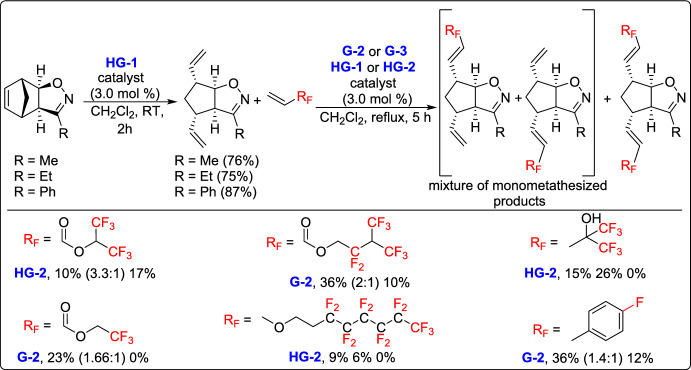
Synthesis of fluorine-containing cyclopentane-fused isoxazolines

In 2021, Kiss and his team made a novel selective functionalization of norbornadiene through nitrile oxide 1,3-dipolar cycloaddition, ROM, and CM strategies [45]. The study began with the selective cycloaddition of nitrile oxides to the C = C bond of norbornadiene, producing cyclopentane-fused isoxazolines. The subsequent ROM process enabled the controlled formation of cyclopentane-fused scaffolds, setting the stage for selective CM. A series of fluorine-containing alkenes as coupling partners were explored to assess substrate scope, as well as catalyst performance. Notably, second-generation Ru-based catalysts (G-2 and HG-2) and third-generation catalyst G-3 exhibited superior efficiency in the CM reactions, while HG-1 failed to afford the desired CM products. These results offered important insight into the chemodifferentiation of olefin bonds and expanded the synthetic toolkit for fluorine-containing bicyclic scaffolds with potential applications in medicinal and materials chemistry (Scheme 20).

**Scheme 21:**
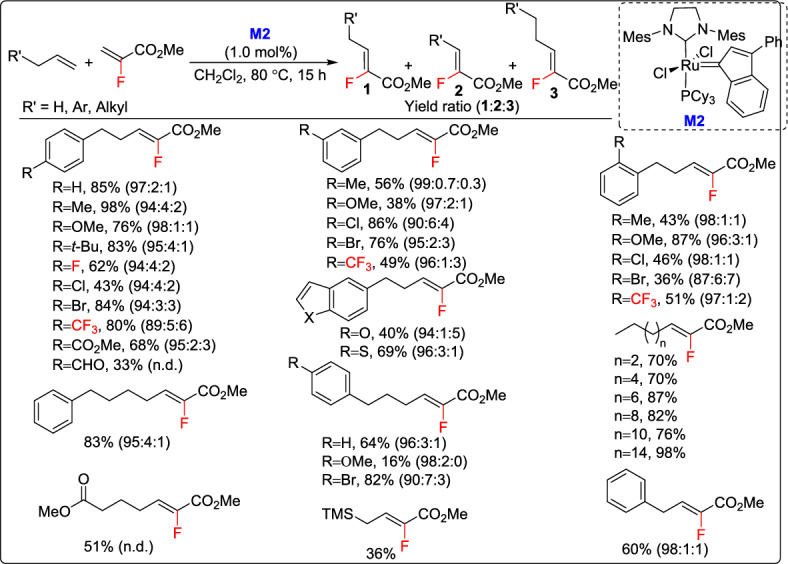
Synthesis of trisubstituted fluoroalkenes through CM

A novel and efficient strategy for the synthesis of trisubstituted fluoroalkenes through CM was reported by Couve-Bonnaire and co-workers, offering a practical and highly stereoselective way to create these valuable fluorinated building blocks [46]. The methodology employed methyl 2-fluoroacrylate as an inexpensive and readily available fluoroalkene source, enabling its selective transformation under ruthenium catalysis, M2. The reaction worked well with over 40 terminal and internal alkenes, producing exclusively the corresponding *Z*-isomers in fair to excellent yields. Notably, an unprecedented turnover number (TON) of up to 175 was achieved, marking a significant milestone in fluoroalkene CM. Using a 1-mol% catalyst loading, this study demonstrated for the first time that such low catalyst amounts could still afford high TONs with fluoroalkenes. However, minor isomerization side-reactions (0–11%) were observed, leading to small proportions of undesired products (Scheme 21). Current research is focused on improving the process by testing more constrained alkenes and alternative fluorinated substrates, which could further enhance the applications of this method in synthetic and medicinal chemistry.

**Scheme 22:**
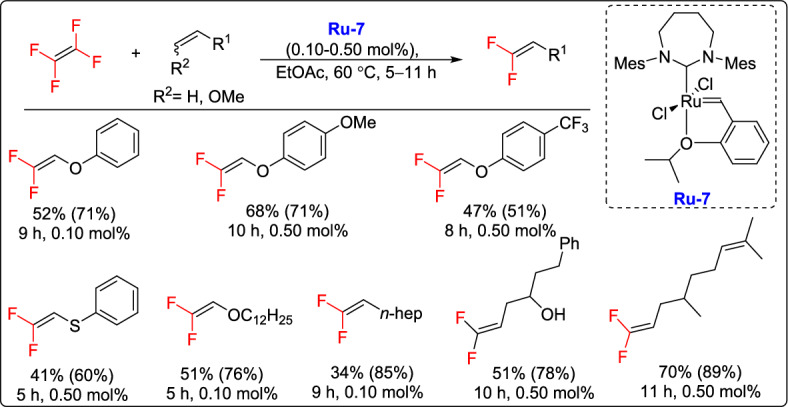
Synthesis of *β*,*β*-difluorovinyl and 1,1-difluoroalk-1-enes, showing isolated yield and ^19^F NMR yield in parentheses

In 2021, Okazoe and co-workers developed a highly efficient method for the CM of TFE with vinyl ethers and vinyl (thio)ethers using an HG-type ruthenium catalyst bearing a seven-membered *N*-heterocyclic carbene ligand (**Ru-7**). This method achieved an impressive catalyst TON value of 4100 under continuous TFE flow. Mechanistic studies supported by DFT calculations showed that the improved reactivity is the result of the unique structure of the expanded NHC ligand, which disrupts stable difluorocarbene intermediates through steric interactions between the mesityl group and fluorine atoms. The reaction provided a reliable way to produce *β*,*β*-difluorovinyl (thio)ethers and 1,1-difluoroalk-1-enes from inexpensive TFE (Scheme 22), offering a practical strategy for accessing valuable fluorinated building blocks. These can serve as versatile intermediates for creating a wide range of fluorine-containing molecules, such as *gem*-difluoromethylene and monofluoroalkene derivatives [47].

**Scheme 23:**
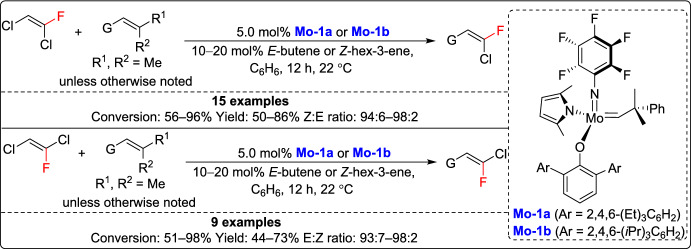
Synthesis of trisubstituted alkenyl fluorides through CM

In 2022, Hoveyda and coworkers created a versatile catalytic approach for the stereodivergent synthesis of trisubstituted alkenyl fluorides, solving long-standing challenges to achieve stereoselective access to these valuable structures. The method started with a CM reaction between two trisubstituted olefins, one of which was a commercially available but underused trihaloalkene, producing a wide variety of stereodefined trisubstituted alkenyl fluorides (Scheme 23). This modular approach allowed access to both *E*- and *Z*-isomers, overcoming the limitations of earlier methods that produced only a single stereoisomer or required complex synthetic steps. The utility of this approach was demonstrated through the synthesis of a fluoronematic liquid crystal component, peptide analogues featuring *E*- or *Z*-amide bond mimics, and some stereoisomers of the anticancer compound, difluororumenic ester. These results highlight the potential of this methodology for diverse applications in medicinal chemistry [48].

**Scheme 24:**
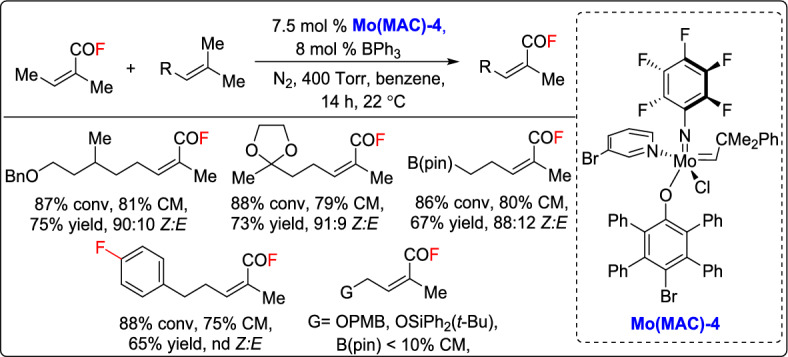
Synthesis of *Z*-trisubstituted *α*-methyl acid fluorides

Recently, the same research group reported a catalytic CM strategy for the synthesis of *Z*-trisubstituted *α*-methyl acid fluorides, a class of compounds that has remained largely unexplored due to the challenging nature of their formation. The presence of an electrophilic acyl fluoride group typically complicates CM, leading to low conversion rates and catalyst decomposition. However, the team successfully addressed this issue by using a Mo(MAC)-4 complex, which enabled efficient *Z*-alkene formation with up to 88% conversion, 75% isolated yield, and 91:9 *Z*/*E* selectivity (Scheme 24). This method allowed the synthesis of various acid fluorides with aryl, acetal, and boronate groups, providing useful fluorinated building blocks for further chemical modifications. The study underscores the significance of optimizing Mo catalyst structures to overcome challenges posed by reactive functional groups in CM reactions [49].

## Ring-Opening Metathesis Polymerization (ROMP) with Fluorinated Derivatives

ROM is another important type of metathesis reaction widely used in synthetic organic chemistry. Its popularity stems from its ability to create diverse polymers and functional materials, which are vital in fields such as material science, polymer chemistry, and biotechnology, to name a few.

**Scheme 25:**
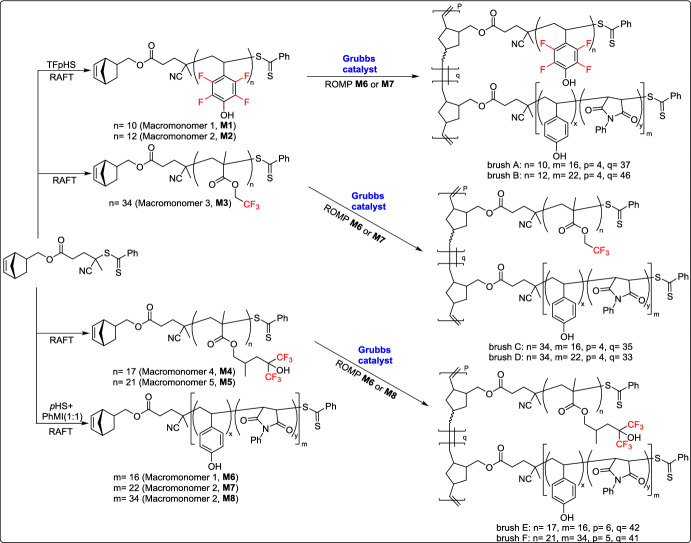
Synthesis of the diblock brush terpolymers

In 2014, Wooley and co-workers reported an approach for the preparation of diblock brush terpolymers (DBTs) with fluorinated methacrylate-based block segments through sequential ROM polymerization [50]. They successfully synthesized nanoscopic cylindrical DBTs with lithographically addressable poly(fluoromethacrylate)(PTFEMA)-based blocks that promote substrate vertical alignment, along with P(pHS-co-PhMI)-based blocks that improved adhesion and allow cross-linking for lithographic pattern formation (Scheme 25). The resulting polymer thin films showed consistent thicknesses and a strong tendency for vertical alignment due to the lower surface energy of the fluorinated structural components and the cylindrical brush architecture. Furthermore, studies on structure–property relationships indicated that the hydrophobic PTFEMA grafts and the higher cross-section of polar grafts enhanced vertical alignment on polar silicon wafer surfaces. These findings highlight the potential of vertically organized block brush copolymers for applications in photolithographic patterning and advanced nanofabrication strategies.

**Scheme 26:**
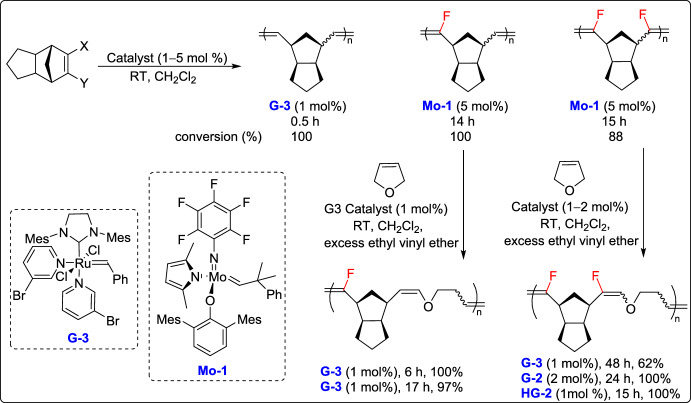
Synthesis of the copolymerization of mono- or difluorinated NBEs; X, Y = H, F

Recently, it was demonstrated that fluoroalkenes, traditionally considered poor substrates for olefin metathesis due to the formation of thermodynamically stable Fischer-type fluorocarbene intermediates, can now be effectively used in alternating ROMP. In this study, it was observed that fluorine substitution at the sp^2^ carbon of norbornene (NBE) derivatives prevents homopolymerization, enabling a highly selective alternating ROMP. To achieve alternating copolymerization using fluorinated NBE, they selected DHF an electron-rich and low-strain cycloalkene as a comonomer. Copolymerization of mono-fluorinated NBEs was carried out using G-3 to target a full degree of polymerization (DP) of 100. In contrast, the full degree of polymerization of difluorinated NBEs was achieved using G-2 and HG-2 (Scheme 26). The alternating nature of the resulting copolymer was confirmed by detailed NMR analyses, and computational studies indicated that the cross-reactivity between fluoroalkenes and enol ethers is driven more by electronic factors than by steric effects. Moreover, the copolymers exhibited tunable degradability under acidic conditions, with monofluorinated enol ethers readily undergoing hydrolysis under mild acidic conditions, while the difluorinated versions displayed enhanced stability. This work highlights the untapped potential of fluoroalkenes in metathesis-based polymer synthesis, providing a new way to create functional fluorinated materials with controlled sequences and degradability [51].

**Scheme 27:**
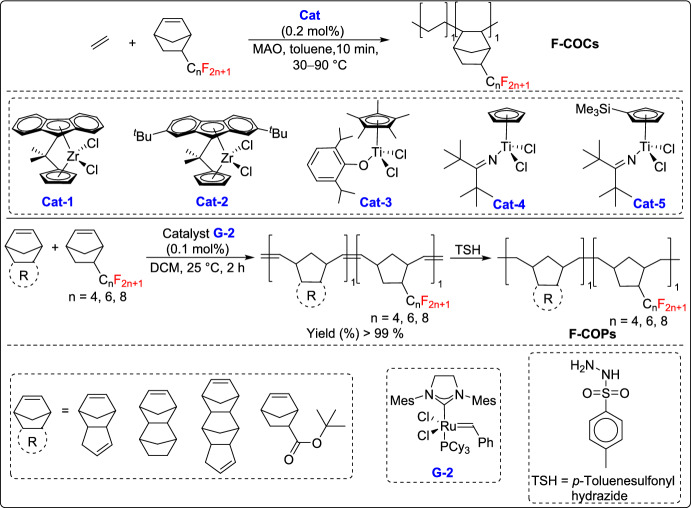
Synthesis of fluorinated cyclic olefin copolymers (F-COCs) and fluorinated cyclic olefin polymers (F-COPs)

In 2025, Jian and coworkers reported an approach for the synthesis of fluorinated cyclic olefin copolymers (F-COCs) and fluorinated cyclic olefin polymers (F-COPs) using coordination-insertion polymerization and ROMP [52]. They successfully synthesized F-COCs and F-COPs with high fluorine contents (up to 48.2 wt%) by incorporating fluorinated norbornene derivatives bearing pendant perfluoroalkyl groups. These materials exhibited excellent dielectric properties, including low dielectric constants (D_k_ = 2.11–2.24) and dielectric losses (D_f_ = 0.0021–0.0031) at 10 GHz. Additionally, these fluoropolymers demonstrated remarkable mechanical properties, such as high toughness with elongation at break up to 779%, along with excellent thermal stability (T_d,5%_ > 427 °C), low hydrophilicity (water uptake < 0.01%), and superior gas separation performance. These characteristics make fluorinated COCs and COPs promising materials for advanced insulating substrates in high-performance electronic and photonic applications (Scheme 27).

## Fluorine-Containing Catalysts for Metathesis Reactions

Recent progress in metathesis chemistry has highlighted the growing importance of fluorine-containing catalysts. This is primarily due to the unique electronic and steric properties that fluorine imparts, enhancing catalyst performance in terms of stability, activity, and selectivity.

**Scheme 28:**
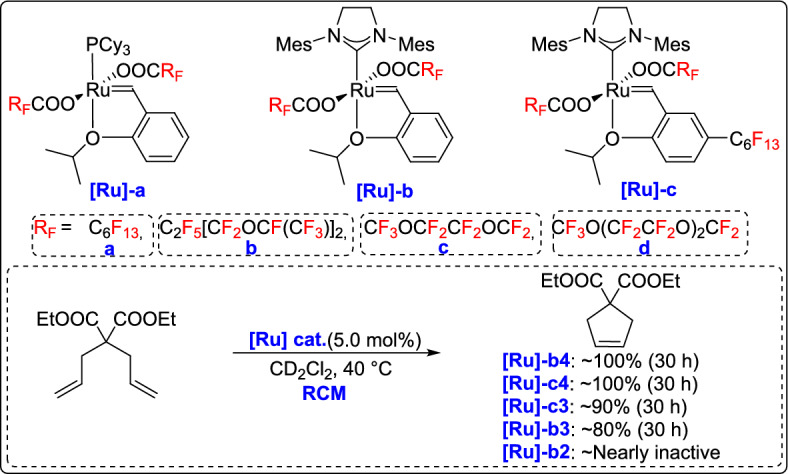
Synthesis of heavy fluorous phosphane-free ruthenium complexes

In 2014, Kvíčala and co-workers developed a series of heavy fluorous, phosphane-free ruthenium complexes based on the HG-2 catalyst by stepwise modification with perfluoroalkylated isopropoxystyrene and two perfluoroalkanoate or perfluoropolyoxaalkanoate ligands. These modifications resulted in high activity in model RCM reactions. A significant discovery was that complexes with linear perfluoropolyether chains performed better, showing higher catalytic activity and fluorophilicity than those with perfluoroalkyl chains. On the other hand, incorporation of branched perfluoropolyether groups led to a marked decrease in activity, likely because of increased steric hindrance around the ruthenium center (Scheme 28) [53].

**Scheme 29:**
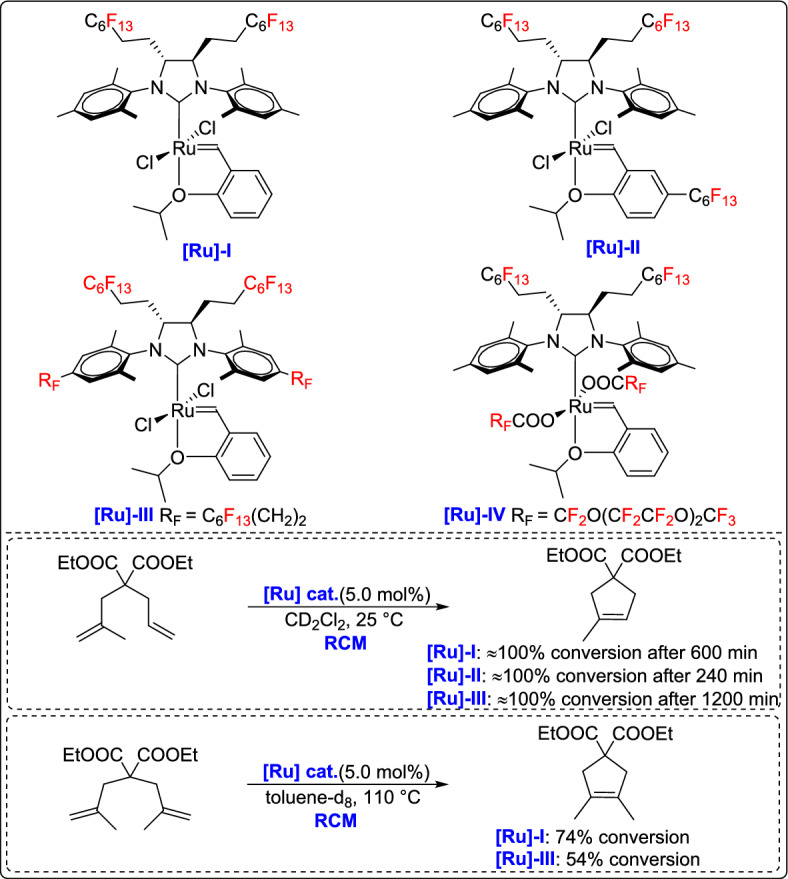
Synthesis of heavy fluorous Hoveyda–Grubbs second-generation precatalysts

The same research group reported the synthesis of fluorous HG-2 precatalysts using the stereoselective addition of polyfluoroalkyllithium to sterically hindered diimines, followed by transformation into NHC-based ruthenium complexes [54]. Among the synthesized complexes, the one bearing four perfluoroalkyl chains retained its fluorous properties even in the active catalytic form, marking the first example of a heavy fluorous alkene metathesis catalyst with such features. These fluorous catalysts exhibited activity and stability comparable to commercial analogues in RCM of tri- and tetra-substituted olefins. Importantly, the heavy fluorous catalyst was efficiently recycled through fluorous-phase separation, demonstrating its potential in sustainable catalysis (Scheme 29).

**Scheme 30:**
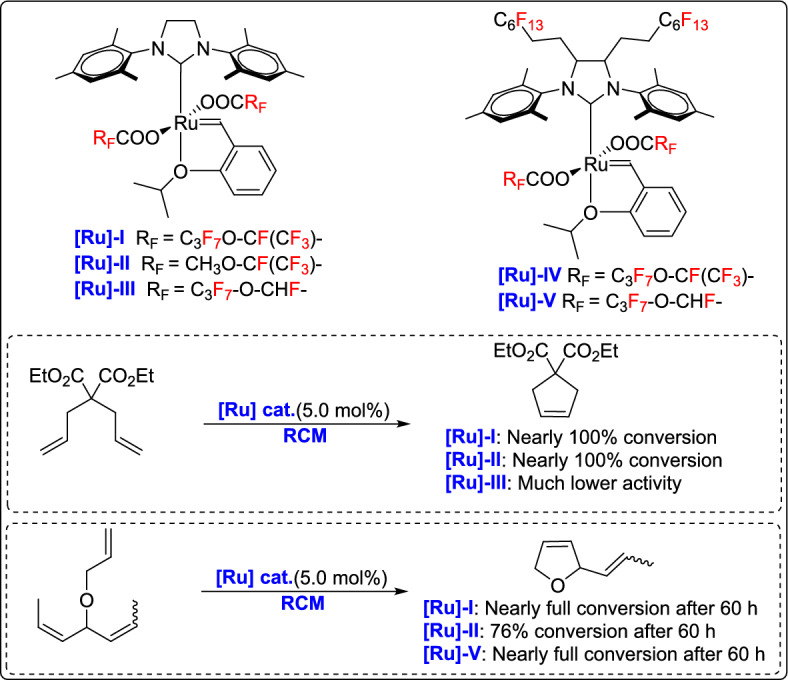
Synthesis of ruthenium complexes modified with chiral racemic per- and poly-fluorooxaalkanoates

In a related study published in 2016, the group developed a new class of ruthenium-based metathesis precatalysts incorporating chiral racemic per- and poly-fluorooxaalkanoate ligands [55]. These complexes were prepared using silver salts of fluorous carboxylic acids, and they were evaluated in model RCM reactions. The study found that catalytic activity declined as steric hindrance increased and the electron-withdrawing nature of the ligand backbone decreased. Notably, the most active precatalyst induced the difficult formation of a tetrasubstituted double bond in the RCM of diethyl dimethallylmalonate. Although the overall activity was lower compared to those of commercial analogues, the performance was sufficient for typical metathesis reactions, offering new perspectives for developing chiral and fluorous catalysts for asymmetric metathesis applications (Scheme 30).

**Scheme 31:**
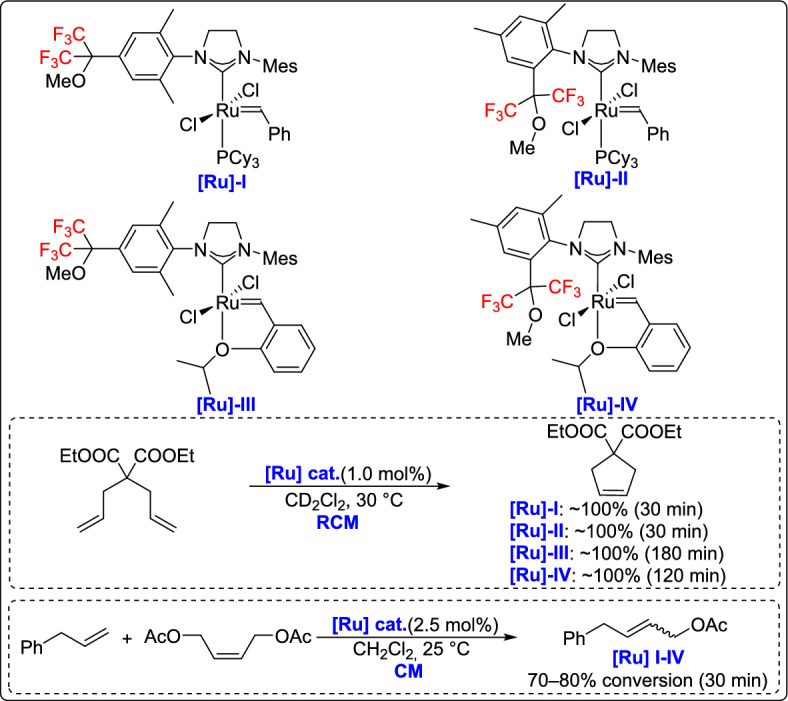
Synthesis of new fluorinated imidazolidinium ruthenium precatalysts

A novel class of fluorinated ruthenium(II) carbene second-generation precatalysts with unsymmetrical NHC ligands was developed for olefin metathesis reactions by the Osipov group [56]. The synthesis involved the creation of fluorinated imidazolidinium salts with a hexafluoroisopropylmethoxy group and mesityl-substituted imidazolidine, resulting in new fluorinated HG-2a catalysts. The Grubbs catalysts showed activity comparable to that of classical second-generation catalysts in olefin metathesis. However, the Hoveyda-type catalysts exhibited latent character in RCM reactions, which was absent when using the traditional second-generation Hoveyda catalyst. This latent behavior could be further enhanced by introducing trifluoromethyl groups on the NHC ligand (Scheme 31).

**Scheme 32:**
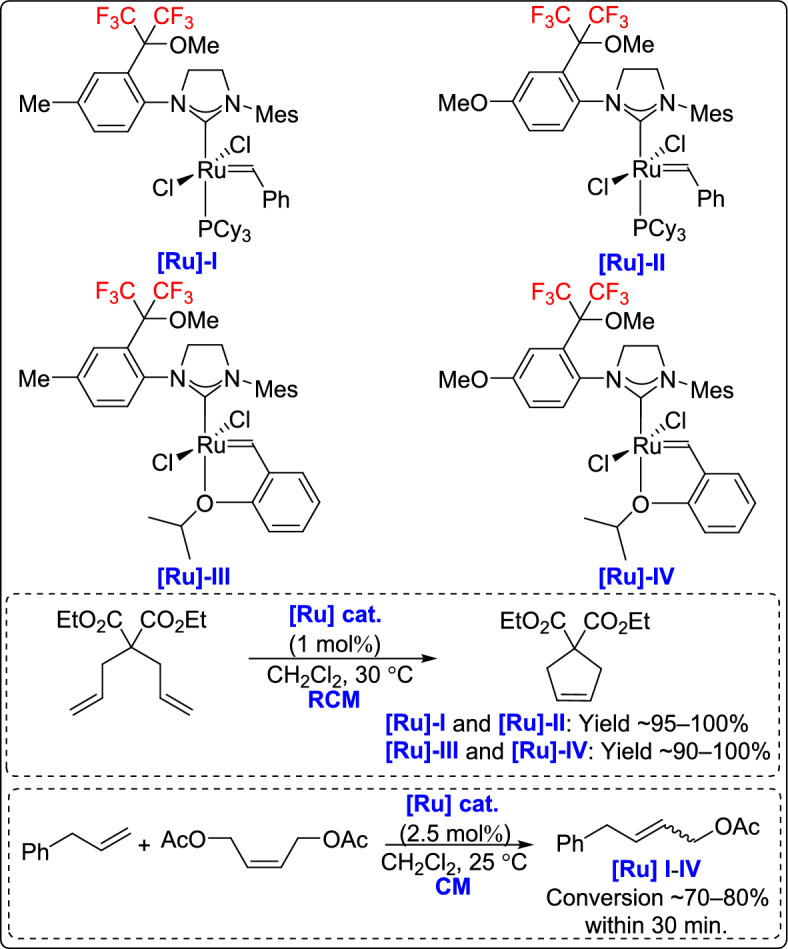
Synthesis of catalysts with fluorinated unsymmetrical fluorinated NHC ligands

A few years later, Osipov and co-workers developed a new olefin metathesis catalyst featuring unsymmetrical fluorinated NHC ligands with a hexafluoroisopropylmethoxy group at the ortho-position of the *N*-aryl substituent [57]. The study explored the effects of mono-ortho-aryl substitution and replacing a para-methyl group with a more electron-donating methoxy group on catalyst activity. Grubbs-type catalysts demonstrated a performance similar to the traditional G-2 catalyst. Hoveyda-type analogues, in turn, exhibited a short induction period before full conversion in the RCM of diallylmalonate, with slower kinetics than the commercial HG-2 catalyst (Scheme 32). These results indicate that rational modification of fluorinated NHC ligands provides a viable strategy for tuning catalyst performance in metathesis transformations.

**Scheme 33.**
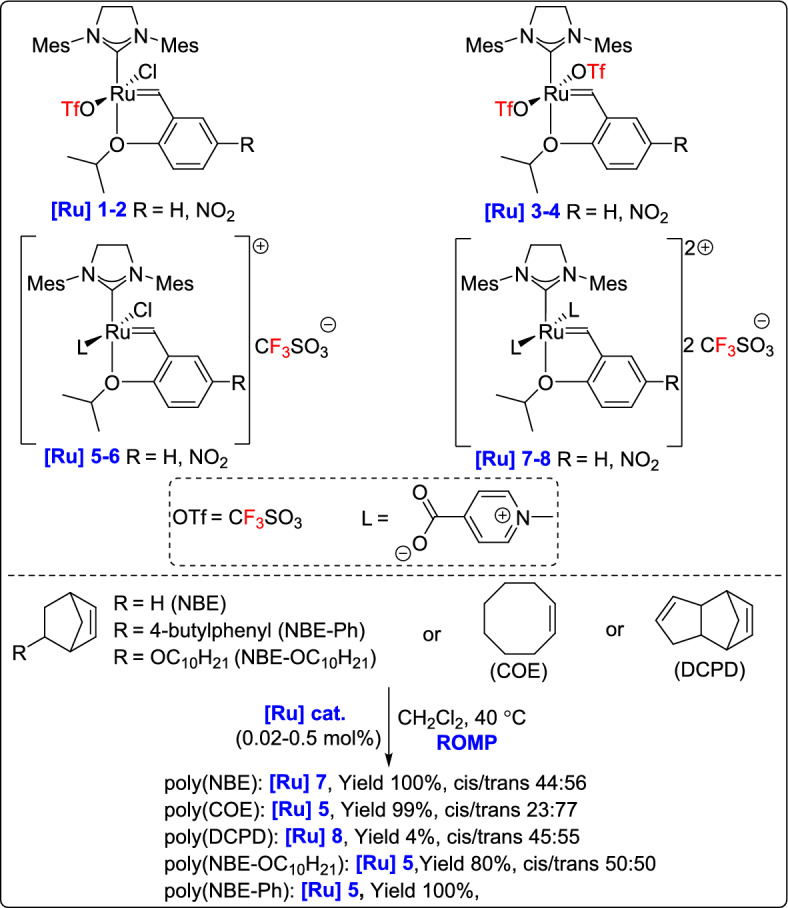
Synthesis of a new class of nitro-substituted ionic Grubbs–Hoveyda complexes

Other researchers have also introduced notable variations in ruthenium systems, Buchmeiser and his team described a new class of nitro-substituted ionic Grubbs–Hoveyda complexes, subjecting them to ROMP under both homogeneous and biphasic conditions [58]. These catalysts displayed high activity toward the ROMP of norbornene-based monomers and cis-cyclooctene under homogeneous conditions. A key advantage of this system is its compatibility with biphasic ROMP in ionic liquids such as [BDMIM^+^][BF_4_^–^], allowing straightforward separation and recycling of the catalyst (Scheme 33). The incorporation of a chain transfer agent enabled the synthesis of polymers with remarkably low ruthenium residues (8–80 ppm) without requiring additional purification steps.

**Scheme 34:**
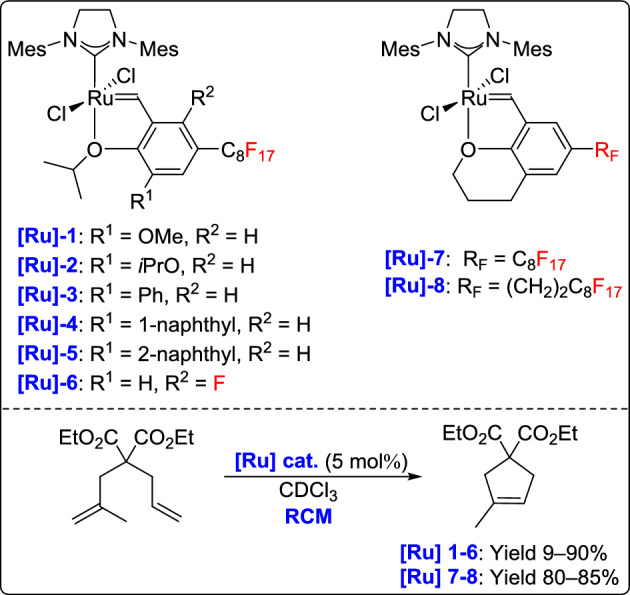
Synthesis of a series of light fluorous Grubbs–Hoveyda second-generation catalysts

In 2017, Matsugi and his team introduced a series of light fluorous HG-2 catalysts through the incorporation of fluorous tags onto the bidentate ligands to enhance catalytic performance. Among the catalysts tested, those featuring a 1-naphthyl group on the ligand demonstrated the highest activity in the RCM of diethyl 2-allyl-2-(2-methylallyl)malonate (Scheme 34). This strategy proved particularly effective during the early stages of the reaction, significantly improving the initial rate of catalysis. However, the approach showed limited success with chromane-type ligands, highlighting the importance of ligand architecture in tuning metathesis efficiency [59].

**Scheme 35:**
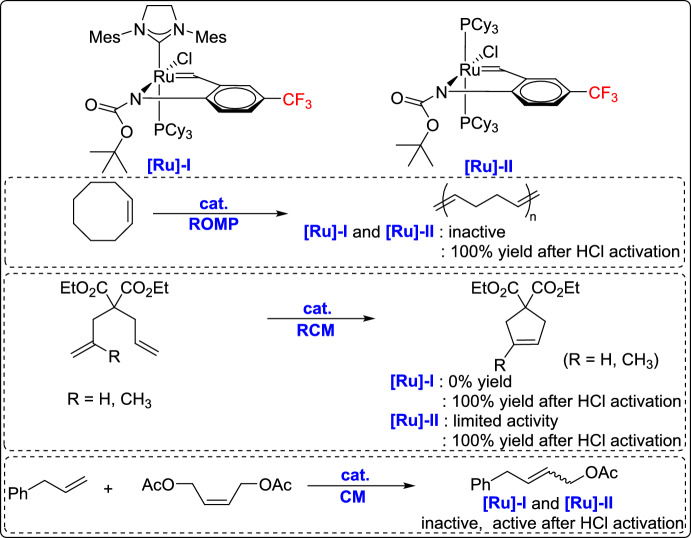
Synthesis of a new carbamato-N-benzylidene ruthenium chelate

Pietraszuk and co-workers contributed to this field by reporting a new carbamato-*N*-benzylidene ruthenium chelates as latent olefin metathesis catalysts through the modification of first- and G-2 catalysts via metathetic exchange with tert-butyl (2-vinylphenyl)carbamates. The chelates featured benzylidene ligands with trifluoromethyl substituents para to the carbamate group. The catalysts were evaluated in various metathesis transformations, including ROMP of cyclooctadiene, RCM of diethyl diallylmalonate and its substituted analogue, as well as CM reactions (Scheme 35). Remarkably, all the complexes exhibited a latent character, remaining completely inactive until treated with an ethereal HCl solution, which protonated the carbamato ligand and modified the ruthenium coordination environment, thereby generating the active species and initiating catalysis [60].

**Scheme 36:**
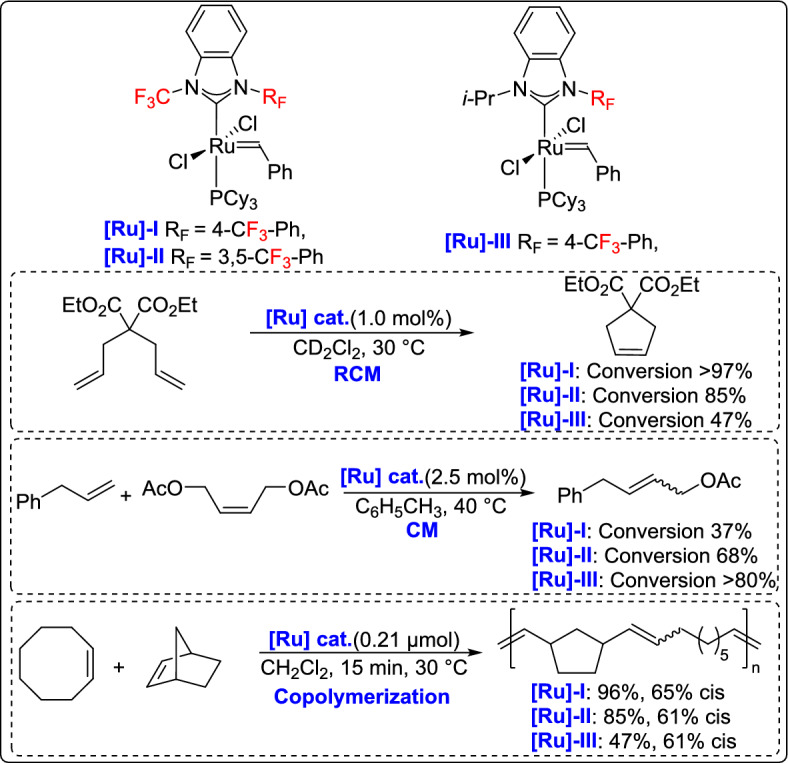
Synthesis of ruthenium metathesis catalysts incorporating unsymmetrical *N*-trifluoromethyl NHC ligands

Togni and his team developed a highly efficient approach to the synthesis of ruthenium metathesis catalysts incorporating unsymmetrical *N*-trifluoromethyl NHC ligands, expanding the scope of metathesis catalysts with distinct electronic properties [61]. These catalysts were evaluated in benchmark olefin metathesis reactions, and they were compared to the standard G-2 catalyst. The *N*-trifluoromethyl catalysts exhibited remarkable selectivity, particularly for the formation of terminal olefins (up to 90%) in the ethenolysis of ethyl oleate. They also showed enhanced selectivity in the alternating copolymerization of cyclooctene and norbornene, highlighting the significant electronic influence of the NHC ligand (Scheme 36).

**Scheme 37:**
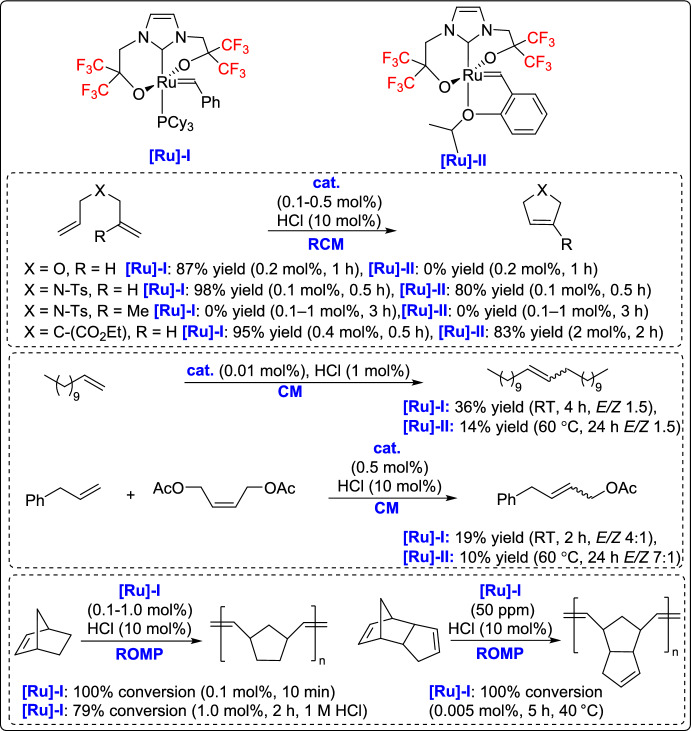
Synthesis of ruthenium complexes with a tridentate anionic bisfluoroalkoxy ligand

More recently, Kajetanowicz and co-workers developed, synthesized, and thoroughly characterized a Hoveyda- and a Grubbs-type ruthenium complex featuring a tridentate anionic bisfluoroalkoxy-carbene ligand, providing new perspectives on catalyst design for olefin metathesis [62]. Although the complexes were initially inactive under standard metathesis conditions, the introduction of HCl activated its catalytic potential, enabling the synthesis of various cyclic olefins and ethers, as well as a stereoregular polynorbornene with an exceptionally high trans-alkene content similar to that of the commercial polymer Norsorex^®^. Structural and computational DFT studies revealed that the complex entered the metathesis cycle but stalled as a stable intermediate, preventing full catalytic turnover. The role of HCl was found to be crucial, inducing dissociation of the fluoroalkoxy units and converting the complex into a reactive species analogous to conventional Grubbs-type catalysts (Scheme 37). This work highlights the importance of catalyst modulation and the potential for designing tailored activation strategies in olefin metathesis.

**Scheme 38:**
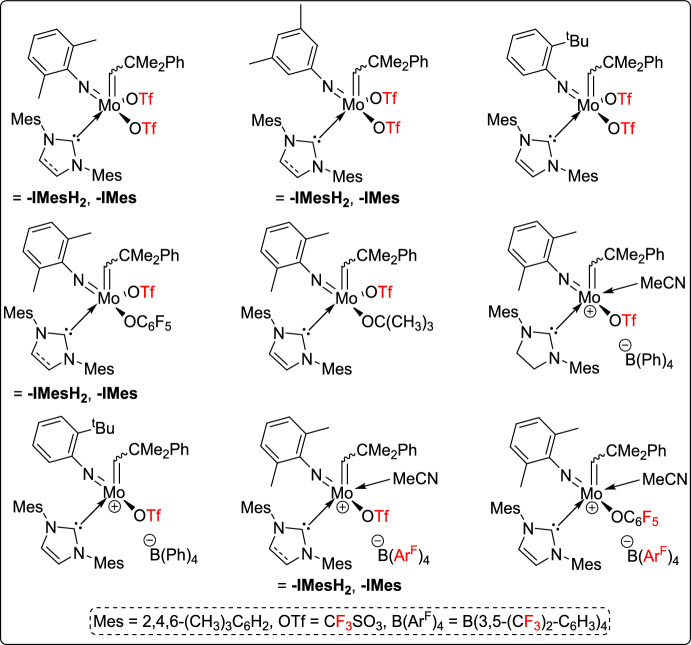
Synthesis of fluorous neutral and cationic molybdenum complexes

Beyond ruthenium, other transition metals have also been explored. For example, Buchmeiser and co-workers investigated a series of neutral molybdenum imido alkylidene NHC bistriflate and monotriflate monoalkoxide complexes, along with cationic molybdenum imido alkylidene triflate complexes (Scheme 38), to explore the mechanism of olefin metathesis using these catalysts. Techniques such as NMR spectroscopy, X-ray crystallography, reaction kinetics, and DFT calculations were employed. The dissociation of an anionic ligand leads to the formation of an intermediary molybdacyclobutane trans to the NHC, which is crucial for catalytic activity. Variations in the NHC, imido, alkoxide, and noncoordinating anions were found to have significant influence on reactivity. Notably, catalysts featuring a single triflate and a single fluorinated alkoxide ligand demonstrated higher reaction rates with 2-methoxystyrene compared to those with two triflate ligands, underscoring the critical role of ligand design in enhancing olefin metathesis efficiency [63].

**Scheme 39:**
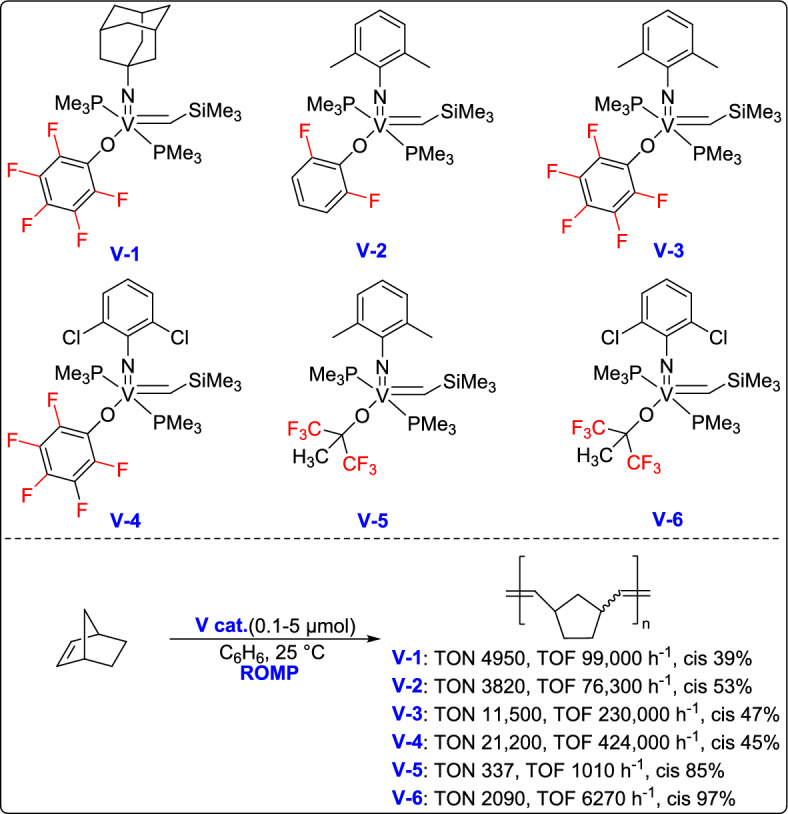
Synthesis of a series of (arylimido)vanadium(V)-alkylidene complexes containing fluorinated aryloxo and alkoxo ligands

In 2015, Hou and Nomura developed a series of (arylimido)vanadium(V)-alkylidene complexes containing fluorinated aryloxo and alkoxo ligands, which demonstrated outstanding catalytic performance in the ROMP of norbornene [64]. These catalysts enabled rapid controlled living ROMP, affording ultrahigh molecular weight polymers with excellent control over polymerization. Notably, the incorporation of fluorinated alkoxo ligands led to highly cis-specific ROMP, with both activity and selectivity further enhanced even at lower temperatures. This work highlights the potential of vanadium-based systems as powerful alternatives to traditional ruthenium and molybdenum ROMP catalysts (Scheme 39).

**Scheme 40:**
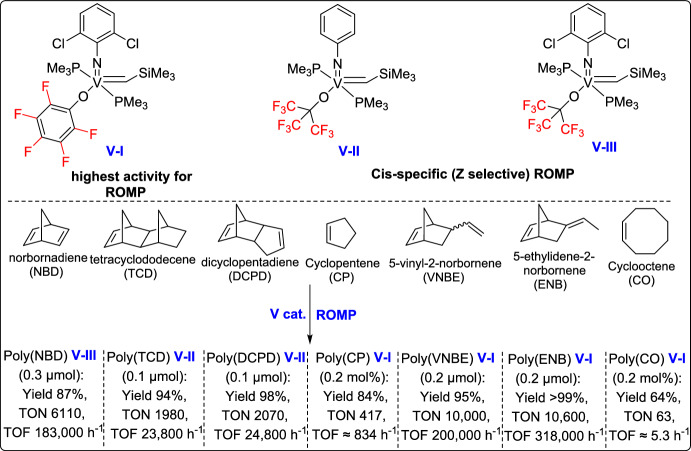
Synthesis of (arylimido)vanadium(V)-alkylidene complexes containing fluorinated aryloxo and alkoxo ligands

One year later, the same research group introduced an efficient strategy for the ROMP of various cycloalkenes using (imido)vanadium(V)-alkylidene catalysts, showing both high catalytic efficiency and excellent cis-selectivity under a broad range of conditions [65]. These catalysts enabled living polymerization processes, producing polymers with low dispersity and controlled molecular weights with remarkable turnover frequencies (TOF; up to 603,000 h⁻^1^). Notably, the activity increased significantly at higher temperatures (50 °C and 80 °C) without affecting the stereoselectivity, which remained at 98%, underscoring the potential of these catalysts for the synthesis of well-defined polymers. Furthermore, the system proved effective, not only for norbornene but also for its derivatives and other strained olefins such as cyclooctene, expanding the scope of applicable monomers under metathesis conditions (Scheme 40).

**Scheme 41:**
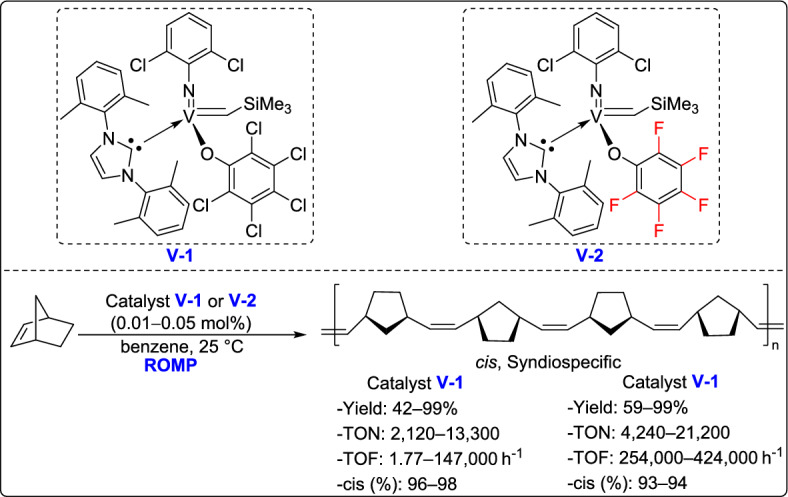
Synthesis of fluorous vanadium(V) arylimido alkylidene complexes

In 2021, Nomura and his team developed a novel class of NHC-coordinated vanadium(V) arylimido alkylidene complexes, which exhibited remarkable activity in ROMP catalysis [66]. These complexes, with the general formula V(N-2,6-Cl₂C₆H₃)(CHSiMe₃)(OC₆X₅)(IXy) [where X = F (3) or Cl (4)], were prepared using specially designed NHC ligands. Interestingly, these complexes demonstrated outstanding catalytic performance in the ROMP of NBE, reaching TOFs as high as 208 s⁻^1^ , and yielding polymers with ultrahigh molecular weights (Mn up to 9.56 × 10^5^) and remarkable cis-syndiotactic stereoregularity up to 98% (Scheme 41). These findings underscore the potential of vanadium(V) alkylidenes as highly active, stereospecific ROMP catalysts, opening new possibilities for the development of advanced polymeric materials.

**Scheme 42:**
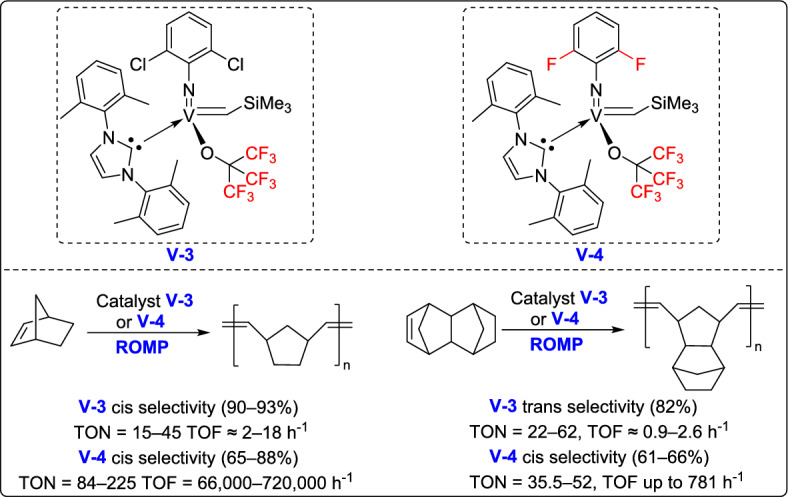
Synthesis of fluorous vanadium(V) arylimido alkylidene complexes

More recently, the same research group reported the synthesis and structural analysis of fluorinated vanadium(V) alkoxide complexes [V(N-2,6-X₂C₆H₃)(CHSiMe₃){OC(CF₃)₃}(NHC)] [X = F (3), Cl (4)], offering new insights into their catalytic behavior in ROMP of NBE and tetracyclododecene (TCD) [67]. Among them, the difluorophenylimido complex displayed lower catalytic activity comparable to dichlorohenylimido systems in the polymerization of NBE. However, the resulting poly(NBE) exhibited moderate cis-selectivity (88%), while the ring-opened poly(TCD) showed even lower stereoselectivity (61–66%), as outlined in Scheme 42. These findings emphasize both the potential and current challenges of fluorinated vanadium alkoxide complexes in achieving stereoselective ROMP, underscoring the need for further optimization of ligand environments to improve selectivity and expand the range of applicable monomers.

## Summary and Outlook

Significant progress has been made in the development and application of fluorinated derivatives in metathesis reactions, particularly in ring-closing metathesis (RCM), cross-metathesis (CM), and ring-opening metathesis polymerization (ROMP) over the past decade. A wide variety of fluorinated cyclic and acyclic compounds with enhanced stability, selectivity, and reactivity have been successfully synthesized, opening new possibilities in pharmaceutical development, advanced materials, and catalytic systems. A key driving force of this progress has been the strategic design of fluorine-modified catalysts, particularly those based on ruthenium, vanadium, and molybdenum. These innovative catalytic systems have significantly expanded the scope of accessible compounds, enabling efficient transformations of even highly challenging substrates. Looking ahead, future advancements in selective and stable fluorinated metathesis catalysts will be key to addressing current limitations in reactivity and stereocontrol. The integration of fluorinated ligands in catalyst frameworks could lead to improved functional group tolerance, enhanced efficiency, and broader substrate applicability. Moreover, the exploration of new fluorinated building blocks and polymers via metathesis strategies holds promise for applications in medicinal chemistry and advanced materials. Further work should prioritize scalable and practical industrial applications to move these methods from laboratory research to large-scale production.

## Data Availability

No datasets were generated or analysed during the current study.
